# The Healthfulness of Entrées and Students’ Purchases in a University Campus Dining Environment

**DOI:** 10.3390/healthcare6020028

**Published:** 2018-03-22

**Authors:** Krista Leischner, Lacey Arneson McCormack, Brian C. Britt, Greg Heiberger, Kendra Kattelmann

**Affiliations:** 1 Health & Nutritional Sciences Department, South Dakota State University, Brookings, SD 57007, USA; Krista.leischner@gmail.com (K.L.); Lacey.McCormack@sdstate.edu (L.A.M.); 2 Journalism & Mass Communications Department, South Dakota State University, Brookings, SD 57007, USA; Brian.Britt@sdstate.edu; 3 Biology & Microbiology, South Dakota State University, Brookings, SD 57007, USA; Greg.Heiberger@sdstate.edu

**Keywords:** campus dining, food purchases, food environment, university dining environment, more healthful, less healthful

## Abstract

The purpose of this study is to determine the availability of “more healthful” (MH) versus “less healthful” (LH) entrée items in the campus dining and if students’ purchases are reflective of what is offered. This is an observational study in which purchases of the available entrée items in the campus dining at South Dakota State University in one academic year were collected and categorized as either MH or LH according to the American Heart Association guidelines. Chi-square tests were used to determine the differences between the proportion of purchased MH and LH versus those available. Odds ratio estimates with 95% confidence limits were used to determine the associations between the demographics and MH and LH purchases. Of the total entrée items available, 15.0% were MH and 85.0% were LH. In the fall, 8.0% of purchases were MH and 92.0% purchases were LH as compared to 8.9% MH and 91.1% LH in the spring. Whites were less likely than non-whites to purchase a MH entrée. Females were two times more likely to choose MH entrées than males. The campus dining offerings and students’ purchases of entrees were primarily LH. Work with campus dining providers to create profitable, yet healthful, dining entrees is needed to improve the healthfulness of offerings.

## 1. Introduction

Obesity has reached record levels and presents a major public health threat. Roughly one in three people nationally in the Unites States are currently obese, and the bodyweight of an average American adult is increasing at a rate of 0.9 kg (1.98 lbs.) per year [1,2]. An obese adult is defined as one with a body mass index (BMI) of greater than or equal to 30; BMI is measured by the ratio of mass in kilograms to height in meters squared [3]. Adult obesity is related to severe health consequences. This disease is detrimental to one’s current health and also leads to complications in future well-being. Possible chronic health concerns resulting from obesity include stroke, sleep apnea, coronary heart disease, hypertension, type 2 diabetes, and dyslipidemia [3,4].

Unhealthy behaviors, such as decreased physical activity levels, increased sedentary time, and poor dietary intake develop during childhood and can continue through adolescence into adulthood [5]. A significant proportion of obese adults were previously obese as young adults (18–25 years) [5,6]. Moreover, young adults who attend college have been shown to gain between 1.8 and 4.1 kg (3.96–9.03 lbs.) annually [1]. Unhealthy weight gain can occur as a result of fluctuations in eating and exercise habits. The often stressful transition from home to college for many young adults can trigger alterations from a normally healthy lifestyle routine to one that promotes the onset of obesity [7]. Such lifestyle modifications, influenced by environmental, occupational, and behavioral changes typically include increased academic stress, increased alcohol intake, decreased physical activity, irregular sleep patterns, and poor dietary behaviors [7,8]. When combined with personal and environmental barriers, these lifestyle modifications can increase one’s risk for the development of obesity. Personal barriers may include a student’s lack of self-control when eating and a lack of motivation to increase healthful habits. An environmental barrier and the focus of this research, is the college dining environment and its relationship with unhealthful food purchases and dietary behaviors of students.

Elements common to the campus dining environment that may negatively affect dietary behaviors include a lack of availability to, and increased prices of, healthful food options and required campus meal plans [9,10]. On-campus dining facilities often offer meal options that cater toward students’ busy school, social, work schedules, and food preferences; thus, many of these options include fast and convenient type foods. Although campus dining can be quick and easy, all too often, unhealthy fast food, oversized portions, and “all-you-can-eat” options are the norm [9,10]. Understandably, this environment can lead to excess energy consumption [10].

While previous studies have linked excess energy consumption to college student weight gain, little is known about the availability of less healthful foods and student purchases. Therefore, the purpose of this study is to determine the healthfulness of the entrée items and purchases of students in the campus dining environment. It is hypothesized that a higher availability of unhealthy foods will reflect a greater percentage of unhealthy foods purchased.

## 2. Materials and Methods

### 2.1. Study Design

This observational study was completed at South Dakota State University and used students’ identification (ID) card data, which included demographic and food item purchasing information from the 2014–2015 school year. This study was conducted according to the guidelines laid down in the Declaration of Helsinki and all procedures involving human subjects were approved by Institutional Review Board, Human Subjects Committee at the university. The study was deemed exempt as the data was collected in a method that human subjects could not be identified, directly or through identifiers linked to the subjects.

First- and second-year college students were required to live in on-campus housing and purchase a campus dining meal plan. Students individually chose their meal plans at the beginning of the school year and used their student ID cards to make all on-campus food purchases during the fall 2014 and spring 2015 semesters; therefore, the data collected from the student ID cards were used to determine entrée purchases through the school year. 

Permanent addresses of students were used to determine the degree of rurality of their home living environment. Students’ zip codes were converted to counties using “Complete Zip Code Totals File” from the United States Census Bureau [11]. Each county was then assigned a Rural-Urban Continuum Code (RUCC) of one through nine, based on the population and proximity to a metro area. Counties assigned an RUCC of 8 or 9 were considered “completely rural or less than 2500 urban population and adjacent to a metro area” and “completely rural or less than 2500 urban population and not adjacent to a metro area”, respectively. Due to the low degree of urbanization around the university where this data was collected, for the purposes of this study, RUCCs 8 and 9 were considered “completely rural” and RUCCs 1–7 were considered “not as rural”. International students were excluded from the study, as their residences could not be assigned an RUCC.

The gender, race, and ethnicity data used in this study were obtained from the Integrated Postsecondary Education Data System (IPEDS) and included gender, Alaskan Native, American Indian, Asian, Black or African American, White, Hispanic or Latino, Multi-Racial, and Unknown. Due to the low frequency of American Indian, Asian, Black or African American, Hispanic or Latino, Multi-Racial, and Unknown, all were combined and categorized as non-white.

### 2.2. Food Items

A list of food items available for purchase was obtained from the campus dining provider and classified as entrées, snack foods and side dishes, drinks, or other. This study is limited to entrées that were purchased as individual items in the eating establishments on campus, which included the following categories of foods: Burger, Entrée Salad No Meat, Entrée Salad with Meat, Meat Entrée, Pizza/Calzone, Salad, Sandwiches/Pitas/Flatbreads/Wraps, Soup/Stew/Chili, Tacos/Nachos, and Vegetarian Entrée. A total of 662 food items were included in the final data set. At the time of the study, all but one eating establishment on campus priced entrees individually. The items from the all-you-can-eat cafeteria were not included in this analysis. At the time of the study, the all-you-can-eat-cafeteria, although open to all students, served primarily athletes.

To access nutrition information regarding the dining options on campus, dining services provider recommended the use of MyFitness Pal (MFP). As such, the online nutrient and calorie tracker was used to assign nutritional information to the majority of the food items. Researchers performed a preliminary search using the foodservice provider’s name plus the specific food item. For those items that did not have the exact match in MFP, the closest best-fit option was chosen and that nutritional information was assigned to the item. If MFP did not have a close best-fit option available, the nutritional information was obtained from the branded vendor’s website. Nutrition information selected for foods was reviewed and approved by a registered dietitian.

Food items were categorized by study personnel as either “more healthful” (MH) if the item met the American Heart Association (AHA) guidelines or “less healthful” (LH) if the item did not. The AHA’s “Recommended Nutritional Standards for Procurement of Foods and Beverages Offered in the Workplace” required all entrées to meet the following limits: less than 500 kcal, less than 480 mg sodium, less than 10% saturated fat, and zero grams of trans fat per serving [12].

### 2.3. Analyses

The number of MH and LH entrées available and frequency purchased in the fall 2014 and spring 2015 semesters was determined. Chi-square tests were used to determine significant differences between the proportion of purchased MH and LH items versus those available during each semester and if the proportion of MH and LH purchases differed between semesters.

A logistic regression was used to determine the relationship between independent variables of demographics of students (completely rural versus not as rural, white versus non-white, and male versus female) and dependent variables of MH and LH purchases in the fall 2014 and spring 2015 semesters. Odds ratio estimates with 95% confidence limits were used to determine the differences between the various demographics and MH and LH purchases in fall versus spring semesters. All analyses were completed in SAS version 9.4 (2012). An alpha level of 0.05 was used for all statistical tests.

## 3. Results

### 3.1. Demographics

The individual entrée purchases of 5177 students were analyzed in the fall, while the entrée purchases of 4613 students were analyzed in the spring. Demographics of the students are shown in Table 1.

### 3.2. More Healthful versus Less Healthfull Purchases

The number of MH and LH entrée items available and purchased in the fall 2014 and spring 2015 semesters are shown in Table 2.

Of the total 662 entrée items available, 15.0% were MH and 85.0% were LH. In the fall, 8.0% of purchases were MH and 92.0% purchases were LH (χ^2^ = 14,028.4, df = 1, *p* < 0.0001). In the spring, 8.9% of purchases were MH and 91.1% were LH (χ^2^ = 7192.1, df = 1, *p* < 0.0001). There was a statistically significant difference in proportion of MH and LH entrée purchases between the fall and spring semesters (χ^2^ = 134.0, df = 1, *p* < 0.0001), with the proportion of MH purchases increasing from 8.0% in the fall to 8.9% in the spring.

### 3.3. Relationships between Student Demographics and Purchases

The relationship between students’ demographics and their MH versus LH food purchases are shown in Table 3. Higher odds ratios indicate that when individuals within the specified groups purchased an entrée, it was more likely to be MH. There was no statistical difference between completely rural and not as rural student purchases in the fall; however, in the spring, for each purchase made by someone from a completely rural population, it was slightly more likely to be a MH entrée (OR = 1.06, 95% CI [1.02, 1.10]). Whites were less likely than non-whites to purchase a MH entrée in both fall and spring semesters (OR = 0.84, 95% CI [0.81, 0.88] in the fall and OR = 0.79, 95% CI [0.75, 0.84] in the spring). Females were two times more likely to choose MH options than males in both the fall (OR = 1.97, 95% CI [1.92, 2.02]) and spring (OR = 2.26, 95% CI [2.20, 2.33]) semesters.

## 4. Discussion

This study examined the availability of LH foods in the campus dining environment and potential relation to purchases of these foods by students. The lack of MH entrée items (15.0%) and overabundance of LH available entrée items (85.0%) suggests the campus dining environment lacks encouragement of healthy dietary behaviors among college students at this university. These results suggest that the environment may influence students’ purchases and that offering a low percentage of MH entrées may result in even fewer MH purchases. These findings are consistent with those reported by Tseng and colleagues in a similar campus study [13]. Tseng reported that of the 314 available entrée items, 88.0% were considered “unhealthful” and the remaining 12.0% were considered “healthful” options as categorized by the Nutrition Environment Measures Survey for campus dining [13].

Campus dining facilities need to run their operations profitably. If healthy food does not sell, foodservice operations have little incentive to offer different options. The findings in this study lay the groundwork for future research to determine why young adults, who are at an increased risk for weight gain, are surrounded by unhealthy foods and how their purchases of these foods may impact their weight over time. It was shown that a large percentage of the foods in the campus dining environment evaluated were considered LH, and the majority of students’ purchases were considered LH. Purchases reflected what was offered, suggesting that, in order to make an impact on college students’ dietary behaviors, the campus dining environment may be important. This impact of the environment on dietary behaviors is supported by the Social Ecological Model (SEM), a program-planning framework suggesting there are different levels of influence (individual, interpersonal, organizational, community, and public policy) on one’s dietary behaviors [14]. Each influence is related to the next, with the broadest influences at the public policy level. Applying the SEM framework to a college students’ food purchases, the campus dining environment falls under the third level (organizational), suggesting the environment strongly impacts a student’s purchases [15]. Findings by Greaney and colleagues address the levels of the SEM influencing college students’ dietary behaviors and stated students identified lack of healthy foods served at dining facilities, easy access to unhealthy foods and fast-food restaurants, and expensive healthful options as barriers to eating healthy in the campus dining environment [16]. In summary, as stated by Horacek, the college dining environment, does not simply feed college students, but has the potential to be highly impactful in the dietary behaviors of college students [17].

Hanks and colleagues at Cornell University have extensively studied adolescents’ healthful food purchases at the high school level and have determined a lunch room that makes healthier options convenient increases purchases of those healthier options [18]. Although a younger age group, these findings may also be applied to the campus dining environment as college students have reported ‘a lack of time’ as a barrier to healthful food choices [19]. Interventions that provide food cost incentives for healthier foods [20] and/or free fruits and vegetables college cafeterias [21] reported modest increase in selection of healthier foods. Nutrition labeling [22,23], nutrition messaging [24], and nutrition information at point of purchase [25,26] have been reported to enhance healthier choices in college dining environments. The type of eating establishment may influence the healthfulness of choices by college campus students. Horacek et al. [27] evaluated the healthfulness of campus eating establishments and reported that among fast food, sit down, cafeteria and take-out establishments that cafeterias were offered healthier options and environment than the other establishments. The entrées evaluated and reported in this paper were offered in franchised kiosk-type eating (fast food), sit down, and take out.

Other notable findings were that non-whites versus whites and females versus males made more MH purchases in both semesters. Females were two times more likely to choose MH options than males. This study was not designed to determine the cause of these differences. However, these differences do support the potential importance of inclusion of culturally appropriate and gender specific programing in interventions developed to improve the dietary choices of college students.

A strength of this study is the analysis of the healthfulness of entrée purchases (373,228 in the fall semester and 247,227 purchases in the spring semester) from a large population of college students. Additionally, because actual food purchases of students were analyzed using sales records, limitations common to self-reported food data were avoided. Assuming the purchased foods were eaten, this form of data collection provides an objective representation of typical eating patterns, with greater accuracy than that of self-reported data for this age group [28]. Lastly, the food purchasing data was collected over a significant period of time (academic school year) versus a shorter period of time, thereby providing a stable representation of purchasing activity rather than one prone to short-term behavioral fluctuations.

Although this study is a strong contributor to the literature addressing the healthfulness of students’ purchases, the results need to interpreted in the context of the limitations. First, this was an observational study collecting the card purchases of students through the academic year and, in order to correlate food purchases with dietary behaviors, it was assumed that the purchased foods were consumed. Likewise, this study did not track students’ off-campus purchases or those at the all-you-care-to-eat dining hall; therefore, only the entrée purchases from on-campus à la carte dining facilities were acknowledged. In addition, the reported nutritional information classifying items as MH and LH was highly dependent on (1) the foodservice provider’s product name, (2) available items in MFP, and (3) the accuracy of the nutritional information in MFP. It was also assumed that the available items did not change between semesters, as foods were offered on fixed or cycle menus. This study occurred in a comprehensive public university that offers bachelors and graduate degrees in a Midwestern state, and 85.4% of the students were white, which may limit the generalizability to other education settings and populations.

## 5. Conclusions

This study addresses the healthfulness of the campus dining options offered in relation to the healthfulness of students’ purchases. Purchases of the students were reflective of what was offered which consisted of primarily less healthful entrée items. Implications for research and practice include the further study of the environment, inclusion of the campus dining providers in the research assessment and intervention and efforts to tailor to the specific audience. Since females tended to have healthier purchases, obesity prevention efforts at the college level should include tailoring towards males. Interventions aiming to improve the dietary behaviors of college students should consider targeting the campus dining environment and public policies (versus only the individual). Future research for obesity prevention interventions should include collaborations with campus dining providers in programming to create profitable, yet healthful, campus dining environments. The relationship between a primarily healthful environment and students’ purchases ought to then be measured to determine if an environment consisting of mainly MH foods correlates with an increase in students’ purchases of MH items.

## Figures and Tables

**Table 1:** Student demographics in the fall and spring semesters.

Demographic	Fall 2014 (*n* = 5177)	Spring 2015 (*n* = 4613)
	% (*n*)	% (*n*)
**Degree of Rurality**		
Completely Rural ^1^	15.8% (818)	15.4% (712)
Not as Rural ^2^	84.2% (4359)	84.6% (3901)
**Race and Ethnicity ^3^**		
White	85.4% (4420)	84.2% (3883)
Non-White	14.6% (757)	15.8% (730)
**Gender ^3,4^**		
Female	52.7% (2600)	52.1% (2249)
Male	47.3% (2334)	47.9% (2068)

^1^ Includes students from counties assigned an RUCC of 8 or 9. RUCC 8 = “completely rural or less than 2500 urban population and adjacent to a metro area” and RUCC 9 = “completely rural or less than 2500 urban population and not adjacent to a metro area.” ^2^ Includes students from counties assigned a RUCC of 1–7. RUCC 1–3 = metro counties and RUCCC 4–7 = more than 2500 urban population. ^3^ Race, ethnicity, and gender were from the Integrated Postsecondary Education Data System. ^4^ Frequencies differ due to missing data.

**Table 2:** More and less healthful entrée items available and purchased.

Entrée Item	Available ^1^ % (*n*)	Purchased	
		Fall % (*n*)	Spring % (*n*)
More Healthful ^2^	15.0% (99)	8.0% (30,010) ^4,5^	8.9% (21,934) ^4,5^
Less Healthful ^3^	85.0% (563)	92.0% (343,218) ^5^	91.1% (225,293) ^5^

^1^ Availability of entrées was assumed to not differ between semesters. ^2^ Defined by the American Heart Association’s Recommended Nutritional Standards for Procurement of Foods and Beverages Offered in the Workplace guidelines as entrée items with less than 500 kcal, less than 480 mg sodium, less than 10% saturated fat, and zero grams trans-fat. ^3^ Defined as foods that did not meet the American Heart Association’s Recommended Nutritional Standards for Procurement of Foods and Beverages Offered in the Workplace guidelines. ^4^ Chi-square test for purchases are significantly different than expected, *p* ≤ 0.001. Expected frequency is weighted based on the proportion of more versus less healthful products purchased. ^5^ Proportion of MH and LH entrée purchases between the fall and spring semesters differed significantly, *p* < 0.0001.

**Table 3:** Relationship between demographics of students and more versus less healthful entrée purchases in the fall and spring semesters.

Demographic	Fall 2014	Spring 2015
	OR (95% CI) ^4^	OR (95% CI)
**Degree of Rurality**		
Completely Rural ^1^	0.99 (0.96–1.03)	1.06 (1.02–1.10)
Versus Not as Rural ^2^		
**Race and Ethnicity ^3^**		
White	0.84 (0.81–0.88)	0.79 (0.75–0.84)
Versus Non-White		
**Gender**		
Female	1.97 (1.92–2.02)	2.26 (2.20–2.33)
Versus Male		

^1^ Includes students from counties assigned a Rural-Urban Continuum Code (RUCC) of 8 and/or 9. RUCC 8 = “completely rural or less than 2500 urban population and adjacent to a metro area.” RUCC 9 = “completely rural or less than 2500 urban population and not adjacent to a metro area.” ^2^ Includes students from counties assigned a RUC code of 1–7. RUCC 1–3 = metro counties and RUCC 4–7 = more than 2500 urban population. ^3^ Compared to Whites; no purchases were reported for Alaskan Natives due to missing data; no comparisons were completed with Pacific Islanders due to the low frequency. ^4^ OR = Odds Ratio and CI = Confidence Interval.
